# Identifying Primary Spontaneous Pneumothorax from Administrative Databases: A Validation Study

**DOI:** 10.1155/2016/1690482

**Published:** 2016-06-08

**Authors:** Eric Frechette, Keegan Guidolin, Ayman Seyam, Yun-Hee Choi, Sarah Jones, J. Andrew McClure, Jennifer Winick-Ng, Blayne Welk, Richard A. Malthaner

**Affiliations:** ^1^Division of Thoracic Surgery, Department of Surgery, Western University, London, ON, Canada N6A 3K7; ^2^Department of Epidemiology and Biostatistics, Western University, London, ON, Canada N6A 3K7; ^3^Division of Pediatric Surgery, Department of Surgery, Western University, London, ON, Canada N6A 3K7; ^4^London Health Sciences Centre, London, ON, Canada N6A 5W9; ^5^Division of Urology, Department of Surgery, Western University, London, ON, Canada N6A 3K7

## Abstract

*Introduction*. Primary spontaneous pneumothorax (PSP) is a disorder commonly encountered in healthy young individuals. There is no differentiation between PSP and secondary pneumothorax (SP) in the current version of the International Classification of Diseases (ICD-10). This complicates the conduct of epidemiological studies on the subject.* Objective*. To validate the accuracy of an algorithm that identifies cases of PSP from administrative databases.* Methods*. The charts of 150 patients who consulted the emergency room (ER) with a recorded main diagnosis of pneumothorax were reviewed to define the type of pneumothorax that occurred. The corresponding hospital administrative data collected during previous hospitalizations and ER visits were processed through the proposed algorithm. The results were compared over two different age groups.* Results*. There were 144 cases of pneumothorax correctly coded (96%). The results obtained from the PSP algorithm demonstrated a significantly higher sensitivity (97% versus 81%, *p* = 0.038) and positive predictive value (87% versus 46%, *p* < 0.001) in patients under 40 years of age than in older patients.* Conclusions*. The proposed algorithm is adequate to identify cases of PSP from administrative databases in the age group classically associated with the disease. This makes possible its utilization in large population-based studies.

## 1. Introduction

Primary spontaneous pneumothorax (PSP) is a relatively frequent clinical entity, reported to be between 6 and 24 cases per 100,000 persons annually [1]. It occurs most commonly in males and has an age distribution peak between 20 and 30 years of age [2, 3]. It is caused by the rupture of lung bulla or blebs. Its treatment varies from simple observation to drainage or surgery in cases of recurrence or massive lung collapse [4]. PSP must be differentiated from SP, which arises from an underlying chronic lung disease, such as COPD, cystic fibrosis, or lymphangioleiomyomatosis, or occurs following chest trauma, as a result of a medical procedure, or some acute medical condition such as status asthmaticus or lung abscess [5]. The peak incidence from a SP has been suggested to arise at a later age, past 55 years of age [6]. Although the initial management of the different types of pneumothorax might be similar, they have different etiology, physiopathology, and principles of long-term treatment [7].

Administrative hospital data are potentially powerful tools, allowing epidemiological study of diseases, but the accuracy of the data must be confirmed. The tenth edition of the International Classification of Diseases (ICD-10-CA) was introduced in Canada in 2000, and it includes codes allowing the identification of patients seeking medical treatment for pneumothorax [8]. A subdivision of the code J93 further differentiates between spontaneous tension pneumothorax, other spontaneous pneumothorax, other pneumothorax, and pneumothorax (unspecified) [9]. Unfortunately, the classification does not clearly allow the identification of PSP, which would be necessary for the development of large population-based studies on the subject using administrative data from emergency room visits and hospital discharge summaries.

The objective of this study was to validate the accuracy of an administrative data algorithm designed to identify PSP cases from the recorded emergency room and hospital discharge summary.

## 2. Materials and Methods

### 2.1. Data Source and Quality

#### 2.1.1. Reference Standard

The research protocol was reviewed and approved by the Health Sciences Research Ethics Board at the University of Western Ontario (00000940-105409). A total of 150 consecutive patients who consulted the emergency department of a single major university hospital serving Southwestern Ontario, Canada, from January 2003 to March 2010 with a main ICD-10-CA diagnosis of pneumothorax (J930, J931, J938, or J939) were identified from the hospital registry recorded in the National Ambulatory Care Reporting System (NACRS). These codes include the complete range of disease severity. Patient charts were abstracted by two independent physicians blinded to the recorded administrative data (KG, AS). The reviewers confirmed or rejected the diagnosis of pneumothorax from the documents contained in the charts, including the radiology reports and images if needed. All cases of pneumothorax were defined as PSP or SP. Where there was disagreement between reviewers, the diagnosis was obtained following a review by a third physician (EF).

#### 2.1.2. Administrative Data

For each patient all information from index date and previous visits Discharge Abstract Database (DAD) and NACRS recorded locally was obtained. The information collected for the analysis included the admission and discharge dates and all ICD-10-CA diagnostic codes. The data from the different visits and databases were pooled for each individual and submitted to the algorithm.

### 2.2. Administrative Data Algorithm

The development of the algorithm was based on the same concepts used clinically to differentiate between PSP and other types of pneumothorax. To identify potential PSP, the algorithm was designed to exclude SP cases using these three steps:

1. Patients with a hospital admission record in DAD in the previous 30 days were excluded unless their main diagnosis was pneumothorax, as they could represent cases of iatrogenic pneumothorax.
2. Patients with a chronic condition associated with SP were excluded. We identified these patients based on all DAD and NACRS recorded locally for 14 conditions present on the index visit date or earlier.
3. We also excluded the patients presenting with an acute condition potentially causing SP in the 30 days prior to the index visit.

The complete list of acute and chronic conditions used in the algorithm, with corresponding codes, is detailed in Table 1. The results obtained from the algorithm and from the chart review were then compared. The results were stratified over two age groups, patients under 40 years and patients 40 years or older, this threshold representing the midpoint of the two distribution peaks for PSP and SP.

### 2.3. Statistical Methods

The chart review results were considered as the gold standard reference for the validation of the proposed algorithm. Comparing the results from the chart review and administrative data algorithm, sensitivity, specificity, positive predictive value (PPV), and negative predictive value (NPV) were calculated, including the 95% confidence interval. Significance levels were obtained from Fisher's exact method using SAS software version 9.3. The kappa statistic was calculated as a measure of agreement between reviewers' results. Additional calculations of sensitivity and specificity were performed to evaluate the ability of the algorithm to define PSP when limiting the diagnosis to patients below different age thresholds set between 30 and 50 years of age.

## 3. Results

Of the 150 patients included in this study, 95 were under the age of 40 years and 55 were aged 40 years or over. The chart review could not identify any evidence of pneumothorax in the charts of 6 patients, while 96% had a pneumothorax corresponding appropriately to the main diagnosis. These included 90 cases of PSP, representing 60% of patients. Eighty-two percent of these (74 cases) were found in patients under 40 years, while 18% were identified in older patients. There was an agreement of 88% between the two reviewers, corresponding to a kappa statistic of 0.76, and a third review was necessary in 18 cases.

The review of the administrative data revealed that the code J939 (pneumothorax, unspecified) was the most commonly used to describe the main diagnosis; however in younger patients, the code J931 was also commonly used. The reason to suspect a SP from the administrative data was most commonly related to an associated chronic condition; however in some patients more than one potential cause was identified. These results are detailed in Table 2.

The ability of the proposed algorithm to identify cases of PSP is detailed in Table 3, which summarizes diagnostic accuracy. Overall, sensitivity of the algorithm for PSP was 94%, being higher in younger patients (97%) than in older patients (81%), *p* = 0.0376. Similarly, a higher PPV was obtained when the algorithm was applied to the younger patients population, 87%, compared to 46% for the patients aged 40 years or more (*p* < 0.0001). The overall specificity of the algorithm was 57%, and no significant difference was identified between the younger and older patient groups. Similarly, there was no significant difference between the two groups NPVs, the overall value estimated to 87%.

**Figure 1:**
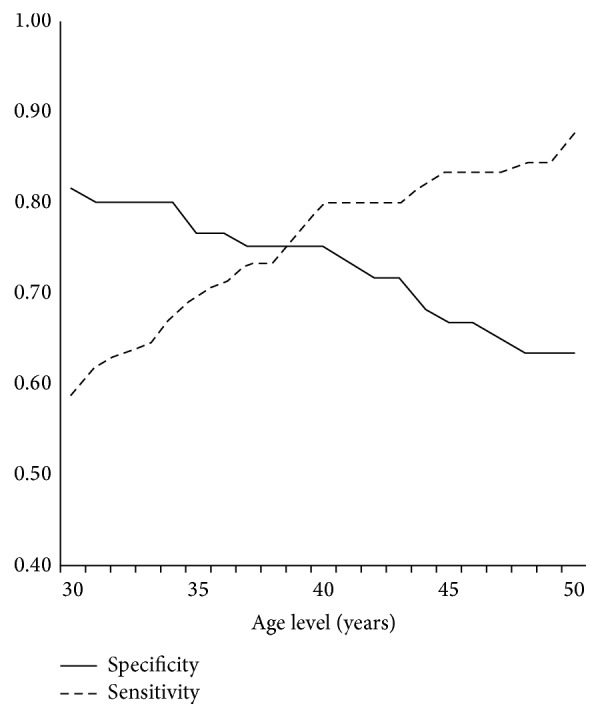
Sensitivity and specificity of the algorithm's modifications applied to the 150 patients and restricting the diagnosis of primary spontaneous pneumothorax to patients below the age level detailed on the axis.

In order to evaluate the appropriateness of the 40-year threshold used in the study, we performed additional modifications on the algorithm for different age levels varying between 30 and 50 years. We calculated the sensitivity and specificity of the algorithm applied to the 150 patients when limiting the diagnosis of PSP to patients younger than each age point. Between the levels of 30 to 50 years, the sensitivity varied from 59% to 88% and the specificity from 82% to 63% (Figure 1).

## 4. Discussion

PSP can happen spontaneously without any underlying cause, usually in young and previously healthy individuals. It differs from SP, which represents a proportion of about half of all pneumothoraces [3, 10–12].

All pneumothoraces will require similar initial management; however, once urgent treatment is completed, the care given to patients presenting with either PSP or SP will differ [6, 13]. Treatment of the underlying cause of SP will be necessary and could include chest wall stabilization, optimization of the COPD or asthma medication, and antimicrobial treatment of pneumonia or lung abscess. For PSP, aside from smoking cessation and recommendation to avoid certain activities, most individuals will require no further treatment, but surgery could be offered to patients who are at a higher risk of recurrence or complications [4, 7, 14].

There is a need for large epidemiological studies on the subject of PSP to help define predictors of recurrence and lead to treatment recommendation tailored to this otherwise healthy and potentially productive population. Population-based studies from the United Kingdom and France have been published in the last few years and have produced significant knowledge of the subject; however, these included both primary and SP. Hallifax and Rahman noted an unexpectedly low proportion of SP (14%) in a report from the French national healthcare database, which has not been fully explained and suggests that a proportion of the cases might not be classified appropriately [15, 16]. In the present review, 54 patients presented with a SP, representing 38% of cases.

The 10th version of the ICD, used in Canada as in many other jurisdictions, does not include a subclassification allowing the identification of the different types of spontaneous pneumothorax. This situation is problematic and limits the ability of researchers to target PSP specifically. In the United States, a different version of the ICD is used, ICD-10-CM (Clinical Modification), which includes a subdivision of the J931 code corresponding to PSP: J9311 [17]. However, it is difficult to anticipate the effect of this subclassification on eventual epidemiological studies because of the presence of three other J93 codes which could be used for PSP. In our study, only 38% of patients were coded J931; the majority (57%) of the charts were labeled with the code J939: pneumothorax, unspecified.

Code-validation is necessary before conducting population-based studies to ensure adequacy of the patient cohorts [18]. In Canada, the Institute for Clinical Evaluative Sciences (ICES) reported in 2006 that the pneumothorax code J93 is amongst the most accurately used (95%) of the main diagnosis codes, and this correlates to the results of this review which identified a miscode rate of only 4% [8]. However, to the authors' knowledge, our report represents the first attempt to differentiate between PSP and SP using hospital data. Defining PSP as only the absence of associated codes for underlying lung disease was not considered sufficient in the design of this study and three exclusion criteria were created with the goal of obtaining a better case definition. Based on the results of the administrative data algorithm, these exclusion criteria identified a significant proportion of patients experiencing SP, particularly in younger patients for which the presence of a chronic condition was responsible for only 42% of cases defined as SP.

PSP is known to occur in a younger population than SP, and multiple studies have suggested that peak incidence occurs between 20 and 30 years of age. When creating the inclusion criteria for a population-based study oriented towards this age group, an option would be to use patient age to minimize the number of misdiagnoses that would be associated with inclusion of older patients. This study's dichotomization of younger versus older patients allowed observation of the effect of this strategy. The ability of the proposed algorithm to accurately identify cases of PSP cases was significantly better when limited to the younger group of patients. However, using this method would exclude cases of true PSP occurring in older patients (16 individuals in this study) and limit the participation to about 82% of potential patients. However, these individuals might also have a nondiagnosed underlying lung condition.

The proposed algorithm demonstrated a good diagnostic accuracy in defining PSP cases amongst patients under 40 years of age. The 97% sensitivity rate found in this age group suggests that almost all PSP cases can be identified with the use of administrative data. And as demonstrated by the 87% PPV, the identification of a PSP case from the algorithm will be correct in a very high proportion of patients. These findings support the use of this method of identification when conducting population-based epidemiological studies of PSP based on administrative data using ICD-10 codes. These findings also suggest that limiting the diagnosis of PSP to the absence of recorded concurrent lung conditions might be insufficient and could be improved by the use of more restrictive exclusion criteria.

The limitations of the proposed algorithm are related to its lower-than-expected specificity. This is a direct consequence of a high number of patients falsely considered to have a PSP, particularly in the older population. This suggests either that the strict criteria used in this study for PSP definition might not be sufficient or, more likely, that the recording of secondary diagnosis into NACRS and DAD databases is occasionally incomplete. Thus, it would be prudent to limit the use of the algorithm to the suggested target population of PSP, as its accuracy might be insufficient when attempting to build a cohort of patients with SP.

## 5. Conclusions

PSP is a clinical entity distinct from SP for which there is a paucity of epidemiological studies. The ICD-10 codes used in most countries do not allow its direct identification. An algorithm was created, which uses the information available in NACRS and DAD to overcome this deficiency. Of 150 patients presenting with a diagnosis of pneumothorax, we validated this algorithm to have a sensitivity of 97% and a PPV of 87% when used in a population of young patients known to be at higher risk of developing a PSP. These results support the use of administrative data to identify patients consulting for PSP and give researchers the tools required to conduct epidemiological studies on large population-based data. Such studies will improve the understanding of the disease, its treatment, and related outcomes.

## Figures and Tables

**Table 1:** Conditions identified as potential causes of secondary pneumothorax with corresponding ICD-10-CA codes.

Condition	ICD-10-CA codes
Chronic obstructive pulmonary disease	J40, J410, J411, J418, J42, J431, J432, J438–J441, J961, J982
Thoracic endometriosis	N808
Pneumocystis	B59
Sarcoidosis	D860–D863, D868, D869
Tuberous sclerosis	Q851
Rheumatoid arthritis	M051–M053, M058–M060, M062–M064, M068, M069, M080
Ankylosing spondylitis	M081, M45
Scleroderma	L940-L941
Ehler-Danlos syndrome	Q796
Marfan syndrome	Q874
Langerhans disease	D760, D763
Cystic fibrosis	E840, E841, E848, E849
Interstitial lung disease	J841, J848, J849
Lung neoplasm	C3400, C3401, C3409–C3411, C3419, C342, C3430, C3431, C3439, C3480, C3481, C3489, C3490, C3491, C3499, C780, C783, D022, D023, D024, D174, D143, D381
Chest trauma^†^	S202–S204, S207, S208, S2100, S2101, S2110, S2111, S2120, S2121, S2170, S2171, S2180, S2181, S2190, S2191, S22000, S22001, S22010, S22011, S22090, S22091, S22100, S22101, S22200, S22201, S22300, S22301, S22400, S22401, S22410, S22411, S22490, S22491, S22500, S22501, S22800, S22900, S230–S235, S240, S2410–S2413, S2418–S2420, S2428, S2438, S2440, S2448, S2458, S2468, S250–S255, S257–S259, S26000, S26001, S26800, S26801, S26810, S26811, S26880, S26881, S26890, S26891, S27000, S27001, S27100, S27101, S27200, S27201, S27300, S27301, S27310, S27311, S27380, S27381, S27390, S27391, S27400, S27410, S27480, S27490, S27500, S27510, S27511, S27580, S27590, S27600, S27601, S27610, S27611, S27680, S27690, S27700, S27701, S27710, S27711, S27780, S27790, S27791, S27800, S27801, S27810, S27811, S27840, S27841, S27850, S27851, S27860, S27861, S27890, S27891, S27900, S27901, S27980, S27981, S280, S281, S2900, S2908, S297–S299, T001, T008–T011, T0180, T0181, T0190, T0191, T0210, T0211, T0270, T0271, T0280, T0281, T0290, T0291, T031, T039, T041, T047, T048, T049, T058–T065, T068, T07, T080, T081, T090, T091, T095, T098, T099, T140–T149, T792, T797, T798, T799
Foreign bodies^†^	T173–T175, T178, T179, T181, T188, T189
Status asthmaticus^†^	J4501, J4511, J4581, J4591
Pneumonia^†^	J120–J123, J128, J129, J13, J14, J150–J160, J168, J180–J182, J188, J189
Lung abscess^†^	J850–J852

^†^Acute conditions (note: all other conditions are considered chronic).

**Table 2:** Distribution of patients with a main diagnosis code of pneumothorax according to age group.

Category	0 to 39 years	40 years and over	Total
Number of patients	95	55	150
*Main diagnosis code*			
J930, spontaneous tension pneumothorax	2	2	4
J931, other spontaneous pneumothorax	46	11	57
J938, other pneumothorax	2	2	4
J939, pneumothorax, unspecified	45	40	85
*Chart review*			
Primary spontaneous pneumothorax	74	16	90
Secondary pneumothorax	17	37	54
Absence of pneumothorax	4	2	6
*Administrative data algorithm*			
Primary spontaneous pneumothorax	83	28	111
Secondary pneumothorax^†^	12	27	39
Suspected etiology: chronic condition	5	18	23
Suspected etiology: acute condition	7	6	13
Suspected etiology: Iatrogenic	3	10	13

^†^More than one suspected etiology may be identified for each patient.

**Table 3:** Diagnostic performance of the administrative data algorithm for the identification of primary spontaneous pneumothorax cases (estimate and 95% confidence interval).

Measure	Total	0 to 39 years	40 years and over	*p*
Sensitivity	94 (88–98)	97 (91–99)	81 (54–96)	0.0376
Specificity	57 (43–69)	48 (26–70)	62 (45–77)	0.4135
Positive predictive value	77 (68–84)	87 (78–93)	46 (28–66)	<0.0001
Negative predictive value	87 (73–96)	83 (52–98)	89 (71–98)	0.6342
